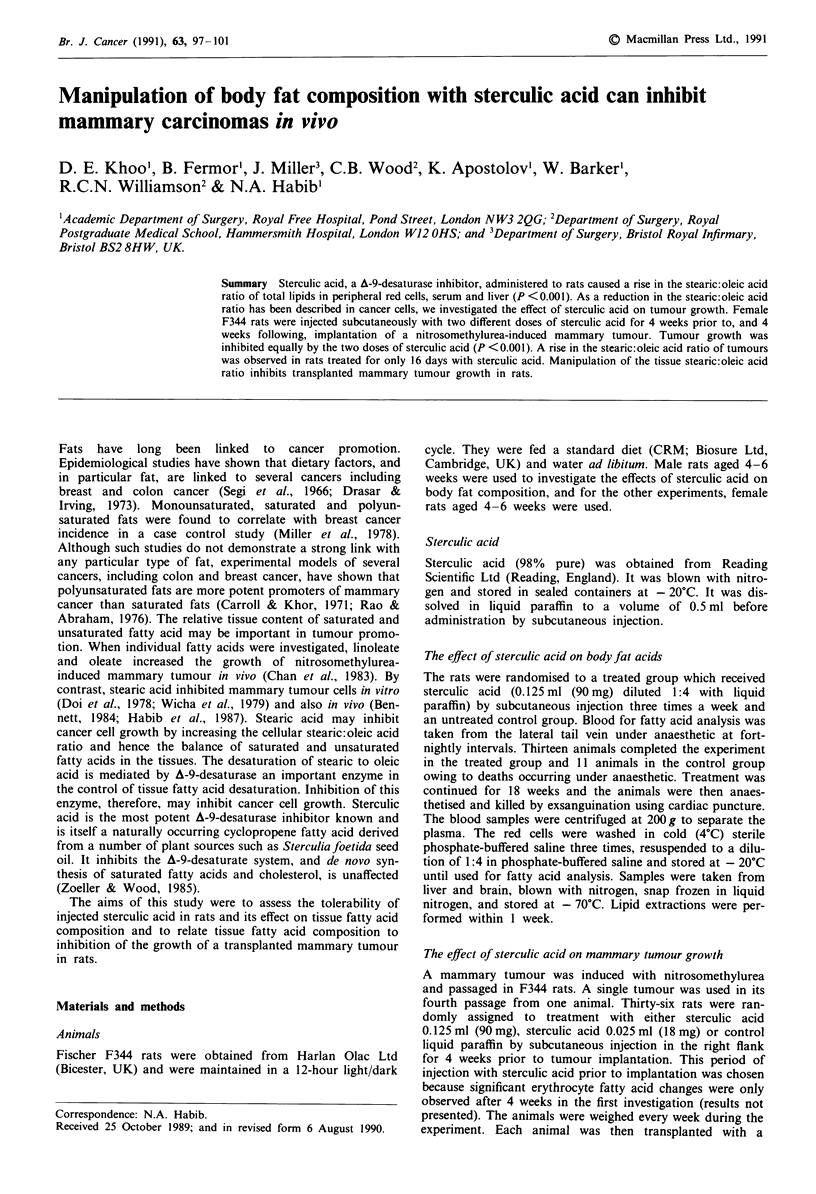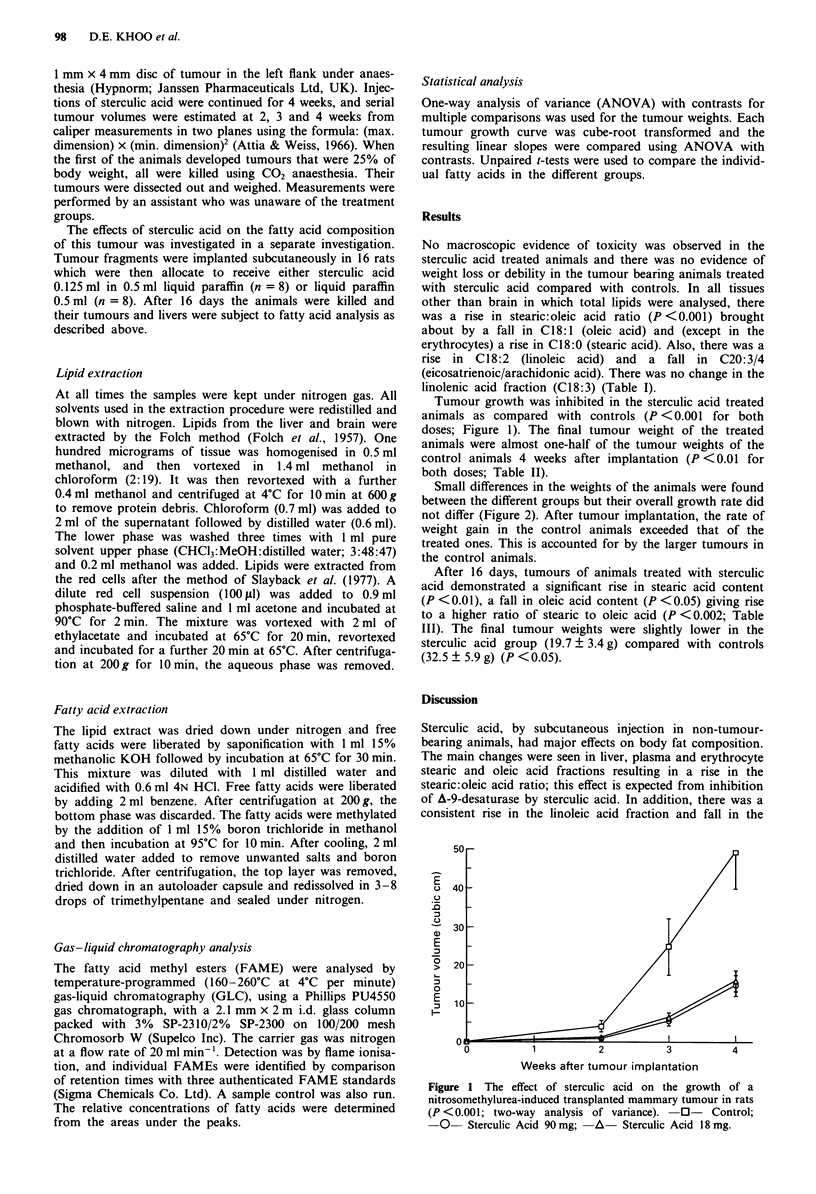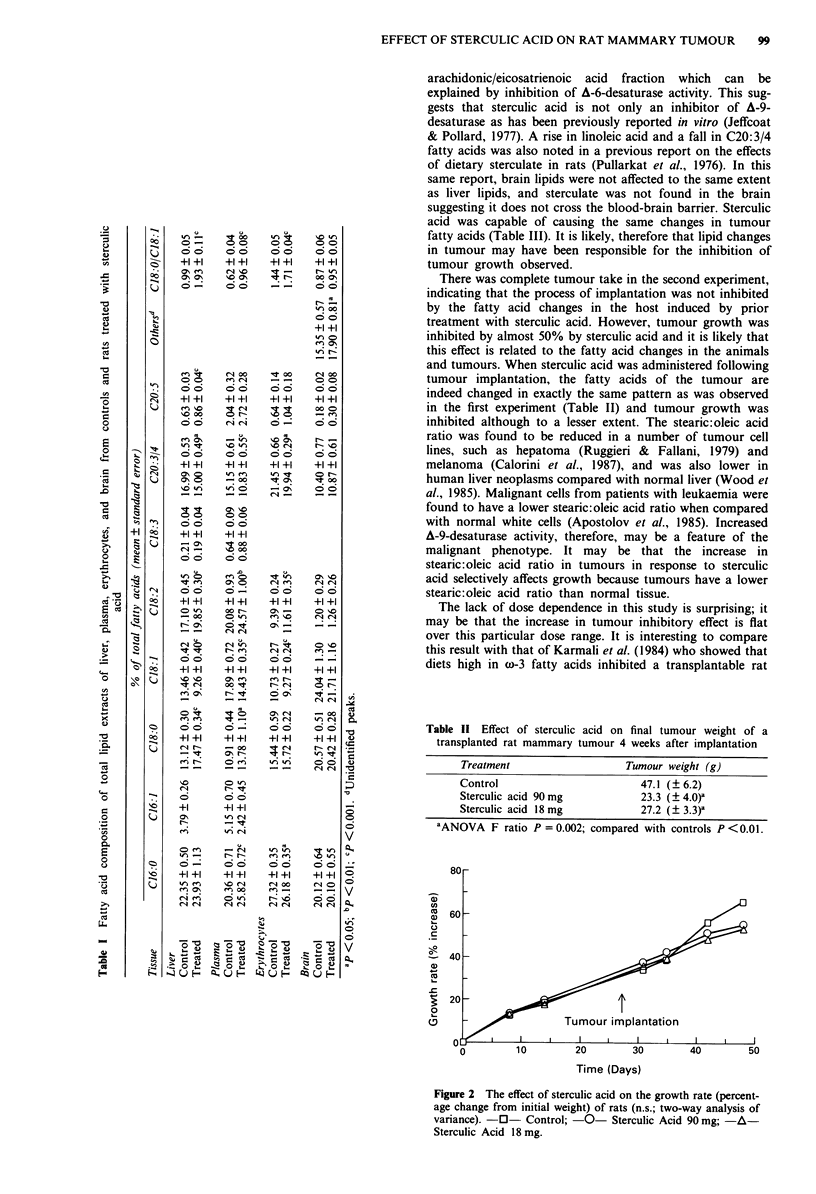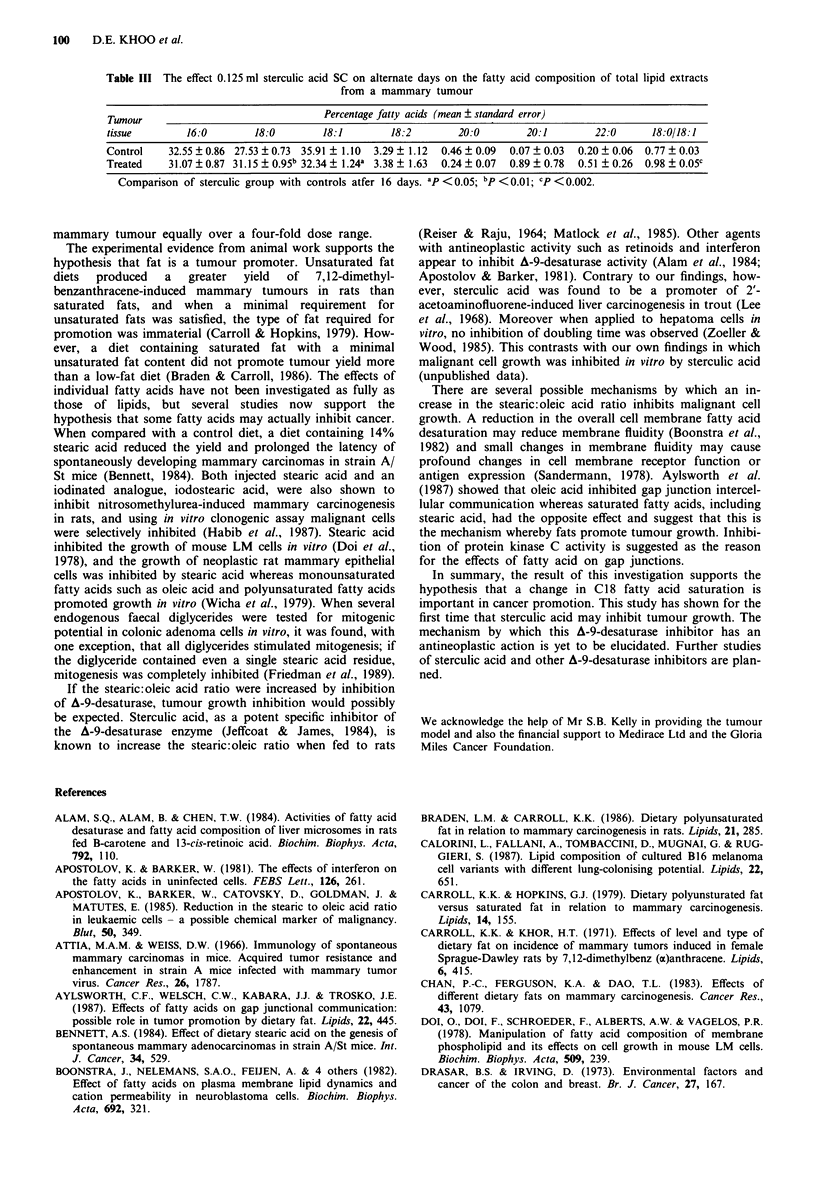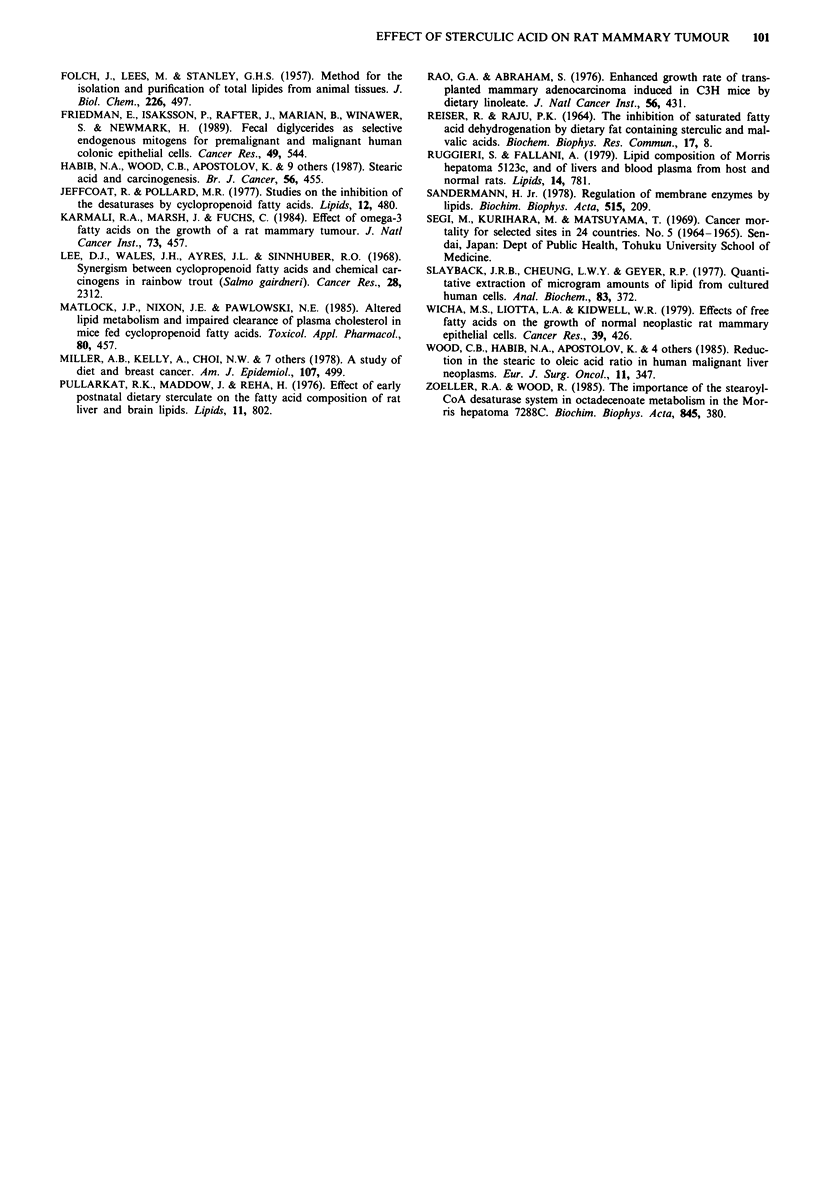# Manipulation of body fat composition with sterculic acid can inhibit mammary carcinomas in vivo.

**DOI:** 10.1038/bjc.1991.20

**Published:** 1991-01

**Authors:** D. E. Khoo, B. Fermor, J. Miller, C. B. Wood, K. Apostolov, W. Barker, R. C. Williamson, N. A. Habib

**Affiliations:** Academic Department of Surgery, Royal Free Hospital, London, UK.

## Abstract

Sterculic acid, a delta-9-desaturase inhibitor, administered to rats caused a rise in the stearic:oleic acid ratio of total lipids in peripheral red cells, serum and liver (P less than 0.001). As a reduction in the stearic:oleic acid ratio has been described in cancer cells, we investigated the effect of sterculic acid on tumour growth. Female F344 rats were injected subcutaneously with two different doses of sterculic acid for 4 weeks prior to, and 4 weeks following, implantation of a nitrosomethylurea-induced mammary tumour. Tumour growth was inhibited equally by the two doses of sterculic acid (P less than 0.001). A rise in the stearic:oleic acid ratio of tumours was observed in rats treated for only 16 days with sterculic acid. Manipulation of the tissue stearic:oleic acid ratio inhibits transplanted mammary tumour growth in rats.


*Br. J. Cancer* (1991), **63**, 97–101 © Macmillan Press Ltd., 1991

### Manipulation of body fat composition with sterculic acid can inhibit mammary carcinomas *in vivo*

D. E. Khoo[1], B. Fermor[1], J. Miller[3], C.B. Wood[2], K. Apostolov[1], W. Barker[1], R.C.N. Williamson[2] & N.A. Habib[1]

[1]*Academic Department of Surgery, Royal Free Hospital, Pond Street, London NW3 2QG;* [2]*Department of Surgery, Royal Postgraduate Medical School, Hammersmith Hospital, London W12 0HS; and* [3]*Department of Surgery, Bristol Royal Infirmary, Bristol BS2 8HW, UK.*

**Summary** Sterculic acid, a Δ-9-desaturase inhibitor, administered to rats caused a rise in the stearic:oleic acid ratio of total lipids in peripheral red cells, serum and liver ($P < 0.001$). As a reduction in the stearic:oleic acid ratio has been described in cancer cells, we investigated the effect of sterculic acid on tumour growth. Female F344 rats were injected subcutaneously with two different doses of sterculic acid for 4 weeks prior to, and 4 weeks following, implantation of a nitrosomethylurea-induced mammary tumour. Tumour growth was inhibited equally by the two doses of sterculic acid ($P < 0.001$). A rise in the stearic:oleic acid ratio of tumours was observed in rats treated for only 16 days with sterculic acid. Manipulation of the tissue stearic:oleic acid ratio inhibits transplanted mammary tumour growth in rats.

Fats have long been linked to cancer promotion. Epidemiological studies have shown that dietary factors, and in particular fat, are linked to several cancers including breast and colon cancer (Segi *et al.*, 1966; Drasar & Irving, 1973). Monounsaturated, saturated and polyunsaturated fats were found to correlate with breast cancer incidence in a case control study (Miller *et al.*, 1978). Although such studies do not demonstrate a strong link with any particular type of fat, experimental models of several cancers, including colon and breast cancer, have shown that polyunsaturated fats are more potent promoters of mammary cancer than saturated fats (Carroll & Khor, 1971; Rao & Abraham, 1976). The relative tissue content of saturated and unsaturated fatty acid may be important in tumour promotion. When individual fatty acids were investigated, linoleate and oleate increased the growth of nitrosomethylurea-induced mammary tumour *in vivo* (Chan *et al.*, 1983). By contrast, stearic acid inhibited mammary tumour cells *in vitro* (Doi *et al.*, 1978; Wicha *et al.*, 1979) and also *in vivo* (Bennett, 1984; Habib *et al.*, 1987). Stearic acid may inhibit cancer cell growth by increasing the cellular stearic:oleic acid ratio and hence the balance of saturated and unsaturated fatty acids in the tissues. The desaturation of stearic to oleic acid is mediated by Δ-9-desaturase an important enzyme in the control of tissue fatty acid desaturation. Inhibition of this enzyme, therefore, may inhibit cancer cell growth. Sterculic acid is the most potent Δ-9-desaturase inhibitor known and is itself a naturally occurring cyclopropene fatty acid derived from a number of plant sources such as *Sterculia foetida* seed oil. It inhibits the Δ-9-desaturate system, and *de novo* synthesis of saturated fatty acids and cholesterol, is unaffected (Zoeller & Wood, 1985).

The aims of this study were to assess the tolerability of injected sterculic acid in rats and its effect on tissue fatty acid composition and to relate tissue fatty acid composition to inhibition of the growth of a transplanted mammary tumour in rats.

### Materials and methods

### *Animals*

Fischer F344 rats were obtained from Harlan Olac Ltd (Bicester, UK) and were maintained in a 12-hour light/dark cycle. They were fed a standard diet (CRM; Biosure Ltd, Cambridge, UK) and water *ad libitum*. Male rats aged 4–6 weeks were used to investigate the effects of sterculic acid on body fat composition, and for the other experiments, female rats aged 4–6 weeks were used.

### *Sterculic acid*

Sterculic acid (98% pure) was obtained from Reading Scientific Ltd (Reading, England). It was blown with nitrogen and stored in sealed containers at − 20°C. It was dissolved in liquid paraffin to a volume of 0.5 ml before administration by subcutaneous injection.

### *The effect of sterculic acid on body fat acids*

The rats were randomised to a treated group which received sterculic acid (0.125 ml (90 mg) diluted 1:4 with liquid paraffin) by subcutaneous injection three times a week and an untreated control group. Blood for fatty acid analysis was taken from the lateral tail vein under anaesthetic at fortnightly intervals. Thirteen animals completed the experiment in the treated group and 11 animals in the control group owing to deaths occurring under anaesthetic. Treatment was continued for 18 weeks and the animals were then anaesthetised and killed by exsanguination using cardiac puncture. The blood samples were centrifuged at 200 *g* to separate the plasma. The red cells were washed in cold (4°C) sterile phosphate-buffered saline three times, resuspended to a dilution of 1:4 in phosphate-buffered saline and stored at − 20°C until used for fatty acid analysis. Samples were taken from liver and brain, blown with nitrogen, snap frozen in liquid nitrogen, and stored at − 70°C. Lipid extractions were performed within 1 week.

### *The effect of sterculic acid on mammary tumour growth*

A mammary tumour was induced with nitrosomethylurea and passaged in F344 rats. A single tumour was used in its fourth passage from one animal. Thirty-six rats were randomly assigned to treatment with either sterculic acid 0.125 ml (90 mg), sterculic acid 0.025 ml (18 mg) or control liquid paraffin by subcutaneous injection in the right flank for 4 weeks prior to tumour implantation. This period of injection with sterculic acid prior to implantation was chosen because significant erythrocyte fatty acid changes were only observed after 4 weeks in the first investigation (results not presented). The animals were weighed every week during the experiment. Each animal was then transplanted with a

Correspondence: N.A. Habib.
Received 25 October 1989; and in revised form 6 August 1990.

1 mm × 4 mm disc of tumour in the left flank under anaesthesia (Hypnorm; Janssen Pharmaceuticals Ltd, UK). Injections of sterculic acid were continued for 4 weeks, and serial tumour volumes were estimated at 2, 3 and 4 weeks from caliper measurements in two planes using the formula: (max. dimension) × (min. dimension)$^2$ (Attia & Weiss, 1966). When the first of the animals developed tumours that were 25% of body weight, all were killed using $CO_2$ anaesthesia. Their tumours were dissected out and weighed. Measurements were performed by an assistant who was unaware of the treatment groups.

The effects of sterculic acid on the fatty acid composition of this tumour was investigated in a separate investigation. Tumour fragments were implanted subcutaneously in 16 rats which were then allocate to receive either sterculic acid 0.125 ml in 0.5 ml liquid paraffin ($n = 8$) or liquid paraffin 0.5 ml ($n = 8$). After 16 days the animals were killed and their tumours and livers were subject to fatty acid analysis as described above.

*Lipid extraction*

At all times the samples were kept under nitrogen gas. All solvents used in the extraction procedure were redistilled and blown with nitrogen. Lipids from the liver and brain were extracted by the Folch method (Folch *et al.*, 1957). One hundred micrograms of tissue was homogenised in 0.5 ml methanol, and then vortexed in 1.4 ml methanol in chloroform (2:19). It was then revortexed with a further 0.4 ml methanol and centrifuged at 4°C for 10 min at 600 *g* to remove protein debris. Chloroform (0.7 ml) was added to 2 ml of the supernatant followed by distilled water (0.6 ml). The lower phase was washed three times with 1 ml pure solvent upper phase ($CHCl_3$:MeOH:distilled water; 3:48:47) and 0.2 ml methanol was added. Lipids were extracted from the red cells after the method of Slayback *et al.* (1977). A dilute red cell suspension (100 μl) was added to 0.9 ml phosphate-buffered saline and 1 ml acetone and incubated at 90°C for 2 min. The mixture was vortexed with 2 ml of ethylacetate and incubated at 65°C for 20 min, revortexed and incubated for a further 20 min at 65°C. After centrifugation at 200 *g* for 10 min, the aqueous phase was removed.

*Fatty acid extraction*

The lipid extract was dried down under nitrogen and free fatty acids were liberated by saponification with 1 ml 15% methanolic KOH followed by incubation at 65°C for 30 min. This mixture was diluted with 1 ml distilled water and acidified with 0.6 ml 4N HCl. Free fatty acids were liberated by adding 2 ml benzene. After centrifugation at 200 *g*, the bottom phase was discarded. The fatty acids were methylated by the addition of 1 ml 15% boron trichloride in methanol and then incubation at 95°C for 10 min. After cooling, 2 ml distilled water added to remove unwanted salts and boron trichloride. After centrifugation, the top layer was removed, dried down in an autoloader capsule and redissolved in 3–8 drops of trimethylpentane and sealed under nitrogen.

*Gas–liquid chromatography analysis*

The fatty acid methyl esters (FAME) were analysed by temperature-programmed (160–260°C at 4°C per minute) gas-liquid chromatography (GLC), using a Phillips PU4550 gas chromatograph, with a 2.1 mm × 2 m i.d. glass column packed with 3% SP-2310/2% SP-2300 on 100/200 mesh Chromosorb W (Supelco Inc). The carrier gas was nitrogen at a flow rate of 20 ml min$^{-1}$. Detection was by flame ionisation, and individual FAMEs were identified by comparison of retention times with three authenticated FAME standards (Sigma Chemicals Co. Ltd). A sample control was also run. The relative concentrations of fatty acids were determined from the areas under the peaks.

*Statistical analysis*

One-way analysis of variance (ANOVA) with contrasts for multiple comparisons was used for the tumour weights. Each tumour growth curve was cube-root transformed and the resulting linear slopes were compared using ANOVA with contrasts. Unpaired *t*-tests were used to compare the individual fatty acids in the different groups.

### Results

No macroscopic evidence of toxicity was observed in the sterculic acid treated animals and there was no evidence of weight loss or debility in the tumour bearing animals treated with sterculic acid compared with controls. In all tissues other than brain in which total lipids were analysed, there was a rise in stearic:oleic acid ratio ($P < 0.001$) brought about by a fall in C18:1 (oleic acid) and (except in the erythrocytes) a rise in C18:0 (stearic acid). Also, there was a rise in C18:2 (linoleic acid) and a fall in C20:3/4 (eicosatrienoic/arachidonic acid). There was no change in the linolenic acid fraction (C18:3) (Table I).

Tumour growth was inhibited in the sterculic acid treated animals as compared with controls ($P < 0.001$ for both doses; Figure 1). The final tumour weight of the treated animals were almost one-half of the tumour weights of the control animals 4 weeks after implantation ($P < 0.01$ for both doses; Table II).

Small differences in the weights of the animals were found between the different groups but their overall growth rate did not differ (Figure 2). After tumour implantation, the rate of weight gain in the control animals exceeded that of the treated ones. This is accounted for by the larger tumours in the control animals.

After 16 days, tumours of animals treated with sterculic acid demonstrated a significant rise in stearic acid content ($P < 0.01$), a fall in oleic acid content ($P < 0.05$) giving rise to a higher ratio of stearic to oleic acid ($P < 0.002$; Table III). The final tumour weights were slightly lower in the sterculic acid group (19.7 ± 3.4 g) compared with controls (32.5 ± 5.9 g) ($P < 0.05$).

### Discussion

Sterculic acid, by subcutaneous injection in non-tumour-bearing animals, had major effects on body fat composition. The main changes were seen in liver, plasma and erythrocyte stearic and oleic acid fractions resulting in a rise in the stearic:oleic acid ratio; this effect is expected from inhibition of Δ-9-desaturase by sterculic acid. In addition, there was a consistent rise in the linoleic acid fraction and fall in the

**Figure 1** The effect of sterculic acid on the growth of a nitrosomethylurea-induced transplanted mammary tumour in rats ($P < 0.001$; two-way analysis of variance). —□— Control; —○— Sterculic Acid 90 mg; —Δ— Sterculic Acid 18 mg.

**Table I** Fatty acid composition of total lipid extracts of liver, plasma, erythrocytes, and brain from controls and rats treated with sterculic acid

| Tissue | % of total fatty acids (mean ± standard error) | | | | | | | | | |
|---|---|---|---|---|---|---|---|---|---|---|
| | *C16:0* | *C16:1* | *C18:0* | *C18:1* | *C18:2* | *C18:3* | *C20:3/4* | *C20:5* | *Others*[d] | *C18:0/C18:1* |
| *Liver* | | | | | | | | | | |
| Control | 22.35 ± 0.50 | 3.79 ± 0.26 | 13.12 ± 0.30 | 13.46 ± 0.42 | 17.10 ± 0.45 | 0.21 ± 0.04 | 16.99 ± 0.53 | 0.63 ± 0.03 | | 0.99 ± 0.05 |
| Treated | 23.93 ± 1.13 | | 17.47 ± 0.34[c] | 9.26 ± 0.40[c] | 19.85 ± 0.30[c] | 0.19 ± 0.04 | 15.00 ± 0.49[a] | 0.86 ± 0.04[c] | | 1.93 ± 0.11[c] |
| *Plasma* | | | | | | | | | | |
| Control | 20.36 ± 0.71 | 5.15 ± 0.70 | 10.91 ± 0.44 | 17.89 ± 0.72 | 20.08 ± 0.93 | 0.64 ± 0.09 | 15.15 ± 0.61 | 2.04 ± 0.32 | | 0.62 ± 0.04 |
| Treated | 25.82 ± 0.72[c] | 2.42 ± 0.45 | 13.78 ± 1.10[a] | 14.43 ± 0.35[c] | 24.57 ± 1.00[b] | 0.88 ± 0.06 | 10.83 ± 0.55[c] | 2.72 ± 0.28 | | 0.96 ± 0.08[c] |
| *Erythrocytes* | | | | | | | | | | |
| Control | 27.32 ± 0.35 | | 15.44 ± 0.59 | 10.73 ± 0.27 | 9.39 ± 0.24 | | 21.45 ± 0.66 | 0.64 ± 0.14 | | 1.44 ± 0.05 |
| Treated | 26.18 ± 0.35[a] | | 15.72 ± 0.22 | 9.27 ± 0.24[c] | 11.61 ± 0.35[c] | | 19.94 ± 0.29[a] | 1.04 ± 0.18 | | 1.71 ± 0.04[c] |
| *Brain* | | | | | | | | | | |
| Control | 20.12 ± 0.64 | | 20.57 ± 0.51 | 24.04 ± 1.30 | 1.20 ± 0.29 | | 10.40 ± 0.77 | 0.18 ± 0.02 | 15.35 ± 0.57 | 0.87 ± 0.06 |
| Treated | 20.10 ± 0.55 | | 20.42 ± 0.28 | 21.71 ± 1.16 | 1.26 ± 0.26 | | 10.87 ± 0.61 | 0.30 ± 0.08 | 17.90 ± 0.81[a] | 0.95 ± 0.05 |

[a]$P < 0.05$; [b]$P < 0.01$; [c]$P < 0.001$. [d]Unidentified peaks.

arachidonic/eicosatrienoic acid fraction which can be explained by inhibition of Δ-6-desaturase activity. This suggests that sterculic acid is not only an inhibitor of Δ-9-desaturase as has been previously reported *in vitro* (Jeffcoat & Pollard, 1977). A rise in linoleic acid and a fall in C20:3/4 fatty acids was also noted in a previous report on the effects of dietary sterculate in rats (Pullarkat *et al.*, 1976). In this same report, brain lipids were not affected to the same extent as liver lipids, and sterculate was not found in the brain suggesting it does not cross the blood-brain barrier. Sterculic acid was capable of causing the same changes in tumour fatty acids (Table III). It is likely, therefore that lipid changes in tumour may have been responsible for the inhibition of tumour growth observed.

There was complete tumour take in the second experiment, indicating that the process of implantation was not inhibited by the fatty acid changes in the host induced by prior treatment with sterculic acid. However, tumour growth was inhibited by almost 50% by sterculic acid and it is likely that this effect is related to the fatty acid changes in the animals and tumours. When sterculic acid was administered following tumour implantation, the fatty acids of the tumour are indeed changed in exactly the same pattern as was observed in the first experiment (Table II) and tumour growth was inhibited although to a lesser extent. The stearic:oleic acid ratio was found to be reduced in a number of tumour cell lines, such as hepatoma (Ruggieri & Fallani, 1979) and melanoma (Calorini *et al.*, 1987), and was also lower in human liver neoplasms compared with normal liver (Wood *et al.*, 1985). Malignant cells from patients with leukaemia were found to have a lower stearic:oleic acid ratio when compared with normal white cells (Apostolov *et al.*, 1985). Increased Δ-9-desaturase activity, therefore, may be a feature of the malignant phenotype. It may be that the increase in stearic:oleic acid ratio in tumours in response to sterculic acid selectively affects growth because tumours have a lower stearic:oleic acid ratio than normal tissue.

The lack of dose dependence in this study is surprising; it may be that the increase in tumour inhibitory effect is flat over this particular dose range. It is interesting to compare this result with that of Karmali *et al.* (1984) who showed that diets high in ω-3 fatty acids inhibited a transplantable rat

**Table II** Effect of sterculic acid on final tumour weight of a transplanted rat mammary tumour 4 weeks after implantation

| *Treatment* | *Tumour weight (g)* |
|---|---|
| Control | 47.1 (± 6.2) |
| Sterculic acid 90 mg | 23.3 (± 4.0)[a] |
| Sterculic acid 18 mg | 27.2 (± 3.3)[a] |

[a]ANOVA F ratio $P = 0.002$; compared with controls $P < 0.01$.

**Figure 2** The effect of sterculic acid on the growth rate (percentage change from initial weight) of rats (n.s.; two-way analysis of variance). —□— Control; —○— Sterculic Acid 90 mg; —Δ— Sterculic Acid 18 mg.

**Table III** The effect 0.125 ml sterculic acid SC on alternate days on the fatty acid composition of total lipid extracts from a mammary tumour

| Tumour tissue | Percentage fatty acids (mean ± standard error) 16:0 | 18:0 | 18:1 | 18:2 | 20:0 | 20:1 | 22:0 | 18:0/18:1 |
|---|---|---|---|---|---|---|---|---|
| Control | 32.55 ± 0.86 | 27.53 ± 0.73 | 35.91 ± 1.10 | 3.29 ± 1.12 | 0.46 ± 0.09 | 0.07 ± 0.03 | 0.20 ± 0.06 | 0.77 ± 0.03 |
| Treated | 31.07 ± 0.87 | 31.15 ± 0.95[b] | 32.34 ± 1.24[a] | 3.38 ± 1.63 | 0.24 ± 0.07 | 0.89 ± 0.78 | 0.51 ± 0.26 | 0.98 ± 0.05[c] |

Comparison of sterculic group with controls atfer 16 days. [a]$P < 0.05$; [b]$P < 0.01$; [c]$P < 0.002$.

mammary tumour equally over a four-fold dose range.

The experimental evidence from animal work supports the hypothesis that fat is a tumour promoter. Unsaturated fat diets produced a greater yield of 7,12-dimethylbenzanthracene-induced mammary tumours in rats than saturated fats, and when a minimal requirement for unsaturated fats was satisfied, the type of fat required for promotion was immaterial (Carroll & Hopkins, 1979). However, a diet containing saturated fat with a minimal unsaturated fat content did not promote tumour yield more than a low-fat diet (Braden & Carroll, 1986). The effects of individual fatty acids have not been investigated as fully as those of lipids, but several studies now support the hypothesis that some fatty acids may actually inhibit cancer. When compared with a control diet, a diet containing 14% stearic acid reduced the yield and prolonged the latency of spontaneously developing mammary carcinomas in strain A/St mice (Bennett, 1984). Both injected stearic acid and an iodinated analogue, iodostearic acid, were also shown to inhibit nitrosomethylurea-induced mammary carcinogenesis in rats, and using *in vitro* clonogenic assay malignant cells were selectively inhibited (Habib *et al.*, 1987). Stearic acid inhibited the growth of mouse LM cells *in vitro* (Doi *et al.*, 1978), and the growth of neoplastic rat mammary epithelial cells was inhibited by stearic acid whereas monounsaturated fatty acids such as oleic acid and polyunsaturated fatty acids promoted growth *in vitro* (Wicha *et al.*, 1979). When several endogenous faecal diglycerides were tested for mitogenic potential in colonic adenoma cells *in vitro*, it was found, with one exception, that all diglycerides stimulated mitogenesis; if the diglyceride contained even a single stearic acid residue, mitogenesis was completely inhibited (Friedman *et al.*, 1989).

If the stearic:oleic acid ratio were increased by inhibition of Δ-9-desaturase, tumour growth inhibition would possibly be expected. Sterculic acid, as a potent specific inhibitor of the Δ-9-desaturase enzyme (Jeffcoat & James, 1984), is known to increase the stearic:oleic ratio when fed to rats (Reiser & Raju, 1964; Matlock *et al.*, 1985). Other agents with antineoplastic activity such as retinoids and interferon appear to inhibit Δ-9-desaturase activity (Alam *et al.*, 1984; Apostolov & Barker, 1981). Contrary to our findings, however, sterculic acid was found to be a promoter of 2′-acetoaminofluorene-induced liver carcinogenesis in trout (Lee *et al.*, 1968). Moreover when applied to hepatoma cells *in vitro*, no inhibition of doubling time was observed (Zoeller & Wood, 1985). This contrasts with our own findings in which malignant cell growth was inhibited *in vitro* by sterculic acid (unpublished data).

There are several possible mechanisms by which an increase in the stearic:oleic acid ratio inhibits malignant cell growth. A reduction in the overall cell membrane fatty acid desaturation may reduce membrane fluidity (Boonstra *et al.*, 1982) and small changes in membrane fluidity may cause profound changes in cell membrane receptor function or antigen expression (Sandermann, 1978). Aylsworth *et al.* (1987) showed that oleic acid inhibited gap junction intercellular communication whereas saturated fatty acids, including stearic acid, had the opposite effect and suggest that this is the mechanism whereby fats promote tumour growth. Inhibition of protein kinase C activity is suggested as the reason for the effects of fatty acid on gap junctions.

In summary, the result of this investigation supports the hypothesis that a change in C18 fatty acid saturation is important in cancer promotion. This study has shown for the first time that sterculic acid may inhibit tumour growth. The mechanism by which this Δ-9-desaturase inhibitor has an antineoplastic action is yet to be elucidated. Further studies of sterculic acid and other Δ-9-desaturase inhibitors are planned.

We acknowledge the help of Mr S.B. Kelly in providing the tumour model and also the financial support to Medirace Ltd and the Gloria Miles Cancer Foundation.

### References

ALAM, S.Q., ALAM, B. & CHEN, T.W. (1984). Activities of fatty acid desaturase and fatty acid composition of liver microsomes in rats fed B-carotene and 13-*cis*-retinoic acid. *Biochim. Biophys. Acta*, **792**, 110.

APOSTOLOV, K. & BARKER, W. (1981). The effects of interferon on the fatty acids in uninfected cells. *FEBS Lett.*, **126**, 261.

APOSTOLOV, K., BARKER, W., CATOVSKY, D., GOLDMAN, J. & MATUTES, E. (1985). Reduction in the stearic to oleic acid ratio in leukaemic cells – a possible chemical marker of malignancy. *Blut*, **50**, 349.

ATTIA, M.A.M. & WEISS, D.W. (1966). Immunology of spontaneous mammary carcinomas in mice. Acquired tumor resistance and enhancement in strain A mice infected with mammary tumor virus. *Cancer Res.*, **26**, 1787.

AYLSWORTH, C.F., WELSCH, C.W., KABARA, J.J. & TROSKO, J.E. (1987). Effects of fatty acids on gap junctional communication: possible role in tumor promotion by dietary fat. *Lipids*, **22**, 445.

BENNETT, A.S. (1984). Effect of dietary stearic acid on the genesis of spontaneous mammary adenocarcinomas in strain A/St mice. *Int. J. Cancer*, **34**, 529.

BOONSTRA, J., NELEMANS, S.A.O., FEIJEN, A. & 4 others (1982). Effect of fatty acids on plasma membrane lipid dynamics and cation permeability in neuroblastoma cells. *Biochim. Biophys. Acta*, **692**, 321.

BRADEN, L.M. & CARROLL, K.K. (1986). Dietary polyunsaturated fat in relation to mammary carcinogenesis in rats. *Lipids*, **21**, 285.

CALORINI, L., FALLANI, A., TOMBACCINI, D., MUGNAI, G. & RUGGIERI, S. (1987). Lipid composition of cultured B16 melanoma cell variants with different lung-colonising potential. *Lipids*, **22**, 651.

CARROLL, K.K. & HOPKINS, G.J. (1979). Dietary polyunsturated fat versus saturated fat in relation to mammary carcinogenesis. *Lipids*, **14**, 155.

CARROLL, K.K. & KHOR, H.T. (1971). Effects of level and type of dietary fat on incidence of mammary tumors induced in female Sprague-Dawley rats by 7,12-dimethylbenz (α)anthracene. *Lipids*, **6**, 415.

CHAN, P.-C., FERGUSON, K.A. & DAO, T.L. (1983). Effects of different dietary fats on mammary carcinogenesis. *Cancer Res.*, **43**, 1079.

DOI, O., DOI, F., SCHROEDER, F., ALBERTS, A.W. & VAGELOS, P.R. (1978). Manipulation of fatty acid composition of membrane phospholipid and its effects on cell growth in mouse LM cells. *Biochim. Biophys. Acta*, **509**, 239.

DRASAR, B.S. & IRVING, D. (1973). Environmental factors and cancer of the colon and breast. *Br. J. Cancer*, **27**, 167.

FOLCH, J., LEES, M. & STANLEY, G.H.S. (1957). Method for the isolation and purification of total lipides from animal tissues. *J. Biol. Chem.*, **226**, 497.

FRIEDMAN, E., ISAKSSON, P., RAFTER, J., MARIAN, B., WINAWER, S. & NEWMARK, H. (1989). Fecal diglycerides as selective endogenous mitogens for premalignant and malignant human colonic epithelial cells. *Cancer Res.*, **49**, 544.

HABIB, N.A., WOOD, C.B., APOSTOLOV, K. & 9 others (1987). Stearic acid and carcinogenesis. *Br. J. Cancer*, **56**, 455.

JEFFCOAT, R. & POLLARD, M.R. (1977). Studies on the inhibition of the desaturases by cyclopropenoid fatty acids. *Lipids*, **12**, 480.

KARMALI, R.A., MARSH, J. & FUCHS, C. (1984). Effect of omega-3 fatty acids on the growth of a rat mammary tumour. *J. Natl Cancer Inst.*, **73**, 457.

LEE, D.J., WALES, J.H., AYRES, J.L. & SINNHUBER, R.O. (1968). Synergism between cyclopropenoid fatty acids and chemical carcinogens in rainbow trout (*Salmo gairdneri*). *Cancer Res.*, **28**, 2312.

MATLOCK, J.P., NIXON, J.E. & PAWLOWSKI, N.E. (1985). Altered lipid metabolism and impaired clearance of plasma cholesterol in mice fed cyclopropenoid fatty acids. *Toxicol. Appl. Pharmacol.*, **80**, 457.

MILLER, A.B., KELLY, A., CHOI, N.W. & 7 others (1978). A study of diet and breast cancer. *Am. J. Epidemiol.*, **107**, 499.

PULLARKAT, R.K., MADDOW, J. & REHA, H. (1976). Effect of early postnatal dietary sterculate on the fatty acid composition of rat liver and brain lipids. *Lipids*, **11**, 802.

RAO, G.A. & ABRAHAM, S. (1976). Enhanced growth rate of transplanted mammary adenocarcinoma induced in C3H mice by dietary linoleate. *J. Natl Cancer Inst.*, **56**, 431.

REISER, R. & RAJU, P.K. (1964). The inhibition of saturated fatty acid dehydrogenation by dietary fat containing sterculic and malvalic acids. *Biochem. Biophys. Res. Commun.*, **17**, 8.

RUGGIERI, S. & FALLANI, A. (1979). Lipid composition of Morris hepatoma 5123c, and of livers and blood plasma from host and normal rats. *Lipids*, **14**, 781.

SANDERMANN, H. Jr. (1978). Regulation of membrane enzymes by lipids. *Biochim. Biophys. Acta*, **515**, 209.

SEGI, M., KURIHARA, M. & MATSUYAMA, T. (1969). Cancer mortality for selected sites in 24 countries. No. 5 (1964–1965). Sendai, Japan: Dept of Public Health, Tohuku University School of Medicine.

SLAYBACK, J.R.B., CHEUNG, L.W.Y. & GEYER, R.P. (1977). Quantitative extraction of microgram amounts of lipid from cultured human cells. *Anal. Biochem.*, **83**, 372.

WICHA, M.S., LIOTTA, L.A. & KIDWELL, W.R. (1979). Effects of free fatty acids on the growth of normal neoplastic rat mammary epithelial cells. *Cancer Res.*, **39**, 426.

WOOD, C.B., HABIB, N.A., APOSTOLOV, K. & 4 others (1985). Reduction in the stearic to oleic acid ratio in human malignant liver neoplasms. *Eur. J. Surg. Oncol.*, **11**, 347.

ZOELLER, R.A. & WOOD, R. (1985). The importance of the stearoyl-CoA desaturase system in octadecenoate metabolism in the Morris hepatoma 7288C. *Biochim. Biophys. Acta*, **845**, 380.